# Diagnostic, Therapeutic, and Prognostic Applications of Artificial Intelligence (AI) in the Clinical Management of Brain Metastases (BMs)

**DOI:** 10.3390/brainsci15070730

**Published:** 2025-07-08

**Authors:** Kyriacos Evangelou, Panagiotis Zemperligkos, Anastasios Politis, Evgenia Lani, Enrique Gutierrez-Valencia, Ioannis Kotsantis, Georgios Velonakis, Efstathios Boviatsis, Lampis C. Stavrinou, Aristotelis Kalyvas

**Affiliations:** 1 Department of Neurosurgery, Attikon University General Hospital, National and Kapodistrian University of Athens, 10679 Athens, Greece; evangeloukyriacos@gmail.com (K.E.); zemperligkos.p@gmail.com (P.Z.); tsspolitis1@gmail.com (A.P.); evgenialani@gmail.com (E.L.); eboviatsis@gmail.com (E.B.); lampis.stavrinou@gmail.com (L.C.S.); 2 Department of Radiation Oncology, University of Toronto, Toronto, ON M5S 1A1, Canada; enrique.gutierrez@uhn.ca; 3 Section of Medical Oncology, Second Department of Internal Medicine, Faculty of Medicine, National and Kapodistrian University of Athens, Attikon University Hospital, 10679 Athens, Greece; ikotsantis@gmail.com; 4 Second Department of Radiology, Faculty of Medicine, National and Kapodistrian University of Athens, Attikon University Hospital, 10679 Athens, Greece; giorvelonakis@gmail.com

**Keywords:** artificial intelligence, brain tumor, brain metastasis, intracerebral metastasis, neurosurgery, neuro-oncology

## Abstract

Brain metastases (BMs) are the most common intracranial tumors in adults. Their heterogeneity, potential multifocality, and complex biomolecular behavior pose significant diagnostic and therapeutic challenges. Artificial intelligence (AI) has the potential to revolutionize BM diagnosis by facilitating early lesion detection, precise imaging segmentation, and non-invasive molecular characterization. Machine learning (ML) and deep learning (DL) models have shown promising results in differentiating BMs from other intracranial tumors with similar imaging characteristics—such as gliomas and primary central nervous system lymphomas (PCNSLs)—and predicting tumor features (e.g., genetic mutations) that can guide individualized and targeted therapies. Intraoperatively, AI-driven systems can enable optimal tumor resection by integrating functional brain maps into preoperative imaging, thus facilitating the identification and safeguarding of eloquent brain regions through augmented reality (AR)-assisted neuronavigation. Even postoperatively, AI can be instrumental for radiotherapy planning personalization through the optimization of dose distribution, maximizing disease control while minimizing adjacent healthy tissue damage. Applications in systemic chemo- and immunotherapy include predictive insights into treatment responses; AI can analyze genomic and radiomic features to facilitate the selection of the most suitable, patient-specific treatment regimen, especially for those whose disease demonstrates specific genetic profiles such as epidermal growth factor receptor mutations (e.g., EGFR, HER2). Moreover, AI-based prognostic models can significantly ameliorate survival and recurrence risk prediction, further contributing to follow-up strategy personalization. Despite these advancements and the promising landscape, multiple challenges—including data availability and variability, decision-making interpretability, and ethical, legal, and regulatory concerns—limit the broader implementation of AI into the everyday clinical management of BMs. Future endeavors should thus prioritize the development of generalized AI models, the combination of large and diverse datasets, and the integration of clinical and molecular data into imaging, in an effort to maximally enhance the clinical application of AI in BM care and optimize patient outcomes.

## 1. Introduction

### 1.1. Overview of Brain Metastases (BMs)

Brain metastases (BMs) are the most common type of intracranial tumors in adults and are three to ten times more common than primary neoplasms [1]. Their prevalence ranges between 10% and 30% in patients with non-CNS (central nervous system) cancer and varies across primary tumor locations and histopathological subtypes [2]. Primary tumors that metastasize to the brain most frequently originate in the lungs (≥50%), breast (15–25%), and skin (melanoma; 5–20%), followed by the testes, kidneys, colon–rectum, and thyroid gland [3]. The rising incidence of BMs is partly attributed to the advancements in systemic therapy that have extended non-CNS cancer patient life expectancy and allowed for greater manifestation of CNS involvement [4]. Advancements in neuroimaging—and particularly magnetic resonance imaging (MRI)—have facilitated early detection and further contributed to the upsurge in BM diagnoses [5].

A multi-step process impelled by malignant cell hematogenous dissemination from their primary extracranial foci describes BM pathophysiology. Circulating tumor cells (CTCs) traverse the blood–brain barrier (BBB) via tumor-derived factor-mediated BBB disruption (e.g., vascular endothelial growth factor, VEGF; matrix metalloproteinases, MMPs) [6]. Metastatic cells interface with the unique neural microenvironment of the brain parenchyma and forward angiogenesis, immune invasion, and disease progression [7]. Vascular permeability dysregulation and astrocytic end-feet disruption promote peritumoral vasogenic edema accumulation that often manifests as neurological deficits, increasing intracranial pressure, significantly contributing to morbidity, and deteriorating patient prognosis [8,9].

### 1.2. Challenges in BM Diagnosis and Treatment

Accurate BM diagnosis is considerably challenging, as their radiological features overlap with those of other intracranial tumors such as gliomas and primary central nervous system lymphomas (PCNSL) [10]. Differentiating between a BM and glioma on conventional MRI becomes even more complex for solitary lesions; advanced imaging modalities such as diffusion-weighted imaging (DWI) and perfusion MRI can facilitate accurate distinction, although not always definitively [11]. Furthermore, BM peritumoral edema can not only mimic glioma infiltration but also complicate tumor boundary delineation and accurate disease burden assessment, influencing surgical and/or radiotherapeutic planning as well [12]. The diagnostic landscape of BMs is additionally complicated by their heterogeneity, considering the diversity of primary tumors (e.g., lung, breast, melanoma) they can arise from [3].

The therapeutic management of BMs is impeded by numerous factors, including the presence of multiple lesions, radioresistance, and systemic therapy response variability [13]. Stemming from different primaries with divergent propensities complicates treatment decisions and warrants multimodal approaches [14]. Radioresistance is a common issue, as tumors adapt to radiation-induced damage and limit the effectiveness of treatments such as stereotactic radiosurgery (SRS) [15]. Moreover, the variability in systemic therapy (chemotherapy, targeted therapy, immunotherapy) response can be unpredictable due to the different molecular backgrounds and genetic profiles that determine tumor behavior [16]. Finally, an intact BBB prevents many treatment agents from reaching BM sites and necessitates alternative strategies to enhance drug delivery [17].

### 1.3. The Role of Artificial Intelligence (AI) in Neuro-Oncology

Artificial intelligence (AI) enables more precise and efficient image analysis to improve BM imaging-based diagnosis. Machine learning (ML)-powered automated segmentation techniques can overcome the limitations of manual assessment and facilitate metastatic lesion identification and delineation on (predominantly) MRI [18]. AI-powered models can detect CNS tumors at early stages, accurately quantify tumor load, and non-invasively predict biomarkers (e.g., metabolic and molecular characteristics) that differentiate BMs from gliomas and PCNSLs based on imaging features [19]. Such tools also aid in detecting subtle peritumoral edema in MRI sequences such as DWI to further differentiate between glioma infiltration and metastasis, a task often challenging even for experienced clinicians [20].

AI is additionally and increasingly utilized for treatment outcome prediction in neuro-oncology. The integration of clinical, imaging, and molecular data can anticipate tumor response to radiotherapy or systemic therapy through predictive AI analytics [21]. Current algorithms analyze imaging features (e.g., lesion shape, texture, vascularity) and correlate those to SRS or fractionated radiotherapy (FRT) outcomes [22]. Analyzing and correlating radiomic features with molecular markers or genomic profiles—such as epidermal growth factor receptor (EGFR) mutations or human epidermal growth factor receptor 2 (HER2) amplification—can contribute to better immuno- and systemic treatment response assessment [23]. AI is capable of collectively scrutinizing its data as more information is accumulated to refine its predictive models and dynamically adjust suggested personalized treatments [24].

### 1.4. Purpose and Scope of This Review

The present review aims to investigate the role of AI in the clinical management of BMs and how the former can contribute to improving diagnosis, therapeutic efficacy, and individualized care. The goal is to spotlight both the benefits and challenges associated with AI technologies in neuro-oncology, identify gaps calling for further research, and serve as a critical overview and resource for healthcare providers and researchers interested in the evolving role of AI in BM management.

## 2. Fundamentals of AI in Oncology and Neuro-Oncology

### 2.1. Overview of AI Technologies

**Figure 1:**
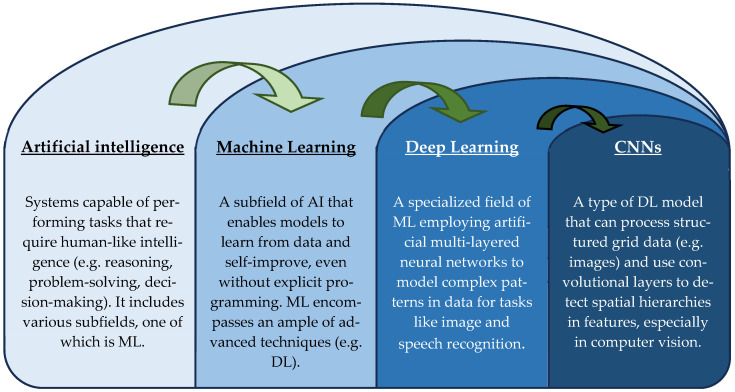
Hierarchical diagram of the nested progression from the broader concept of AI to specialized CNNs. AI = artificial intelligence; CNNs = convolutional neural networks; DL = deep learning; ML = machine learning.

AI in medicine has flourished thanks to ML, one of its most important branches. ML involves the automation of decision-making using algorithms that can predict outcomes based on input data and without manual programming [25]. While ML models can learn and apply their experience in predictive analytics (e.g., forecasting disease course in oncology), treatment personalization (e.g., optimal chemotherapy regimen identification), and automated image analysis (e.g., detection of abnormalities in radiological images) [26], even more advanced subsets such as deep learning (DL) can analyze much more complex data; DL excels at handling large datasets—such as those associated with radiological images, genomic/molecular profiles, and electronic patient records—and can even extract insights from clinical notes via natural language processing (NLP) [27]. Its greatest contribution to radiology and diagnostics is associated with so-called convolutional neural networks (CNNs), algorithms that use convolutional layers for automatic spatial feature extraction from images [28]. Figure 1 illustrates the nested hierarchy of AI, ML, DL, and CNNs.

AI emerged from rudimentary symbolic reasoning in the mid-20th century and evolved through early rule-based systems (e.g., CASNET, MYCIN) in the 1970s [29,30]. Such systems supported clinical decision-making but were limited by computational constraints and static knowledge bases. The so-called “AI Winters”—periods of diminished interest and funding in artificial intelligence—occurred in the late 1970s (the “first AI Winter”) and again in the late 1980s to early 1990s (the “second AI Winter”) [31]. Despite this, the field of clinical informatics continued to progress, supported by the development of expert systems such as INTERNIST-1 and DXplain [32,33]. A resurgence in medical AI began in the early 2000s with the advent of ML, DL, and CNNs, which have since transformed specialties including radiology, dermatology, and pathology [34]. Its potential in clinical decision-making was exemplified by the DeepQA system of IBM Watson in 2011 and FDA-approved DL applications (such as Arterys in 2017) with clinical variability in diagnostic radiology [35,36]. AI-supported approaches nowadays encompass real-time endoscopic lesion detection [37], predictive analytics in oncology [38], and intraoperative applications during robotic-assisted surgery [39].

### 2.2. Radiomics and Radiogenomics

Quantitative feature extraction from imaging data can provide insights into tumor heterogeneity, vascular patterns, and diffusion metrics in the context of the growing field of radiomics [40]. Clinical outcomes such as treatment response have been shown to correlate with features such as lesion texture, shape, and intensity variations [41]. Contemporary advanced imaging—and especially dynamic contrast-enhanced MRI—can be used to evaluate vascular patterns indicative of tumor aggressiveness and metastatic risk [42]. Additionally, tissue diffusivity, an indicator of malignant cellularity and tumor microenvironment characteristics, can be quantified using diffusion-weighted imaging (DWI) and apparent diffusion coefficient (ADC) maps in tumors such as glioblastomas, where lower ADC values typically correspond to higher tumor cell densities [43].

Radiogenomics, on the other hand, refers to the coalescence of imaging data and genomic/molecular tumor characteristics and drives the evolution of more personalized treatments in oncology [44]. Such innovations include the integration of distinct imaging features in various cancers with their molecular profiles: HER2-positive breast cancer, for instance, typically demonstrates increased vascularization and texture alterations on MRI that can be detected by ML and correlate with its underlying molecular changes [45]. Similarly, EGFR mutations in non-small cell lung cancer (NSCLC) have been linked to metabolic activity alterations that can be traced on positron emission tomography (PET) scans [46]. This integration of radiomic features with genomic data can provide a comprehensive and individualized disease overview, improving diagnostic accuracy, patient-specific treatment tailoring, and, ultimately, prognosis [47].

### 2.3. AI Algorithms in Oncology

Oncology has significantly benefited from CNNs in terms of early detection and diagnosis in numerous fields. Algorithms trained on datasets regarding mammography and ultrasound (e.g., DDSM; Digital Database for Screening Mammography) [48], lung cancer on computed tomography (CT) scans (e.g., IQ-OTH/NCCD; Iraq Oncology Teaching Hospital/National Center for Cancer Diseases) [49], and microscopy images for leukemia (e.g., ALL-IDB; Acute Lymphoblastic Leukemia Image Database and C-NMC) [50] have demonstrated excellent tumor classification accuracies. Similar models have shown improved accuracy in colorectal [51] and thyroid [52] cancer classification based on histopathological and ultrasound images, respectively, while GoogLeNet has reached almost 90% sensitivity for bladder cancer detection on cystoscopy images [53] and prostate cancer classification on MRI [54]. CNNs have also performed well using optical coherence tomography (OCT) imaging in ovarian cancer (e.g., ConvLSTM) [55] and CT scans for liver cancer segmentation (e.g., ESP-UNet; Edge Strengthening Parallel UNet) [56].

**Figure 2:**
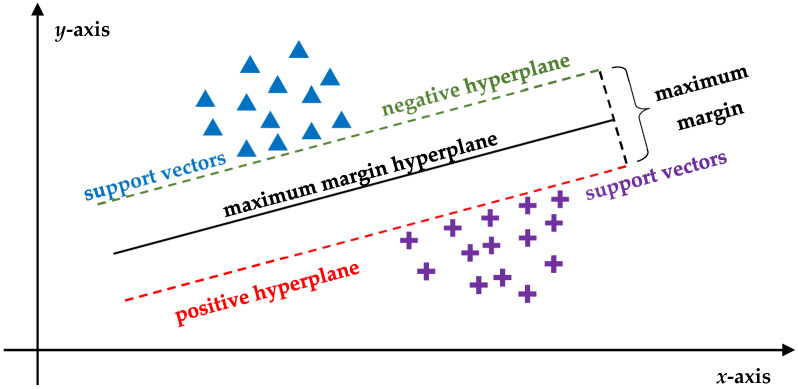
Simplified scatter plot outlining a hypothetical SVM-based binary classification. The two types of points represent different types of lesions (e.g., blue triangles → benign versus purple crosses → malignant) and the axes correspond to specific features (e.g., *y*-axis → size and *x*-axis → density). The positive and negative hyperplanes indicate the thresholds for classifying a lesion as benign or malignant, respectively, while the maximum margin hyperplane is positioned in a way that maximizes the pooled perpendicular distance between the two classes. SMVs aim to identify this line to optimize the separation between the classes and minimize the risk of misclassification. To do so, they focus on the points closest to the maximum margin hyperplane (support vectors), which influence the position of the line. The greatest possible distance between the support vectors of the two classes is called the maximum margin; greater maximum margins indicate better models that are less likely to misclassify new data. SVM = support vector machine.

In addition to CNNs, ML-based algorithms such as support vector machines (SVMs) have been widely tested in radiomics-based feature classification in oncology and have been deemed particularly effective in distinguishing malignant from benign lesions based on imaging-derived radiomic data (e.g., MRI, CT, PET) [57]. Simply put, an SVM is a type of algorithm that learns how to tell two categories apart by drawing the best possible dividing line (or surface)—known as hyperplane—between them based on the input data. It selects this line in a way that maximizes the separation between the two groups, making the classification as accurate as possible [58] (Figure 2).

SVMs have been extensively integrated into oncological genomics, with applications in cancer classification and subtyping, biomarker and signature identification, drug discovery, driver gene discovery, and gene interaction studies [59]. SVM-derived models have been explored for various malignancies, such as breast cancer, where they managed to classify patients based on histological features, genetic profiles, and clinical characteristics with high accuracy, facilitating prompt diagnosis and therapy [60].

## 3. AI Applications in BM Diagnosis

### 3.1. Lesion Detection and Diagnostic Imaging Segmentation

The first step in diagnosing brain lesions with the help of AI is the partitioning of the digital images into multiple segments, a process known as segmentation [61]. DL algorithms used for image segmentation require extensive training on large datasets with pixel-wise labeling [62]; multiple such annotated databases have thus been developed for AI model training and validation in BMs and predominantly include MRI scans with additional clinical information [63]. Some of the largest datasets were published last year and include the New York University (NYU) and NYU Langone Health (NYUMets) database (2367 lesions) [64], the University of California San Francisco Brain Metastases Stereotactic Radiosurgery (UCSF-BMSR) dataset (5136 lesions) [65], and a Research Data Deposit (RDD)-available dataset (no. RDDB2022116272) including 10,388 metastases [66].

The burdensome and time-consuming task of manual brain lesion segmentation—especially when multiple metastases are present—predisposes to inaccuracies in tumor border delineation, is limited by intra- and interobserver variability, and cannot be easily reproduced [67]. Given the need for effective computer-assisted lesion detection and segmentation in neuro-oncology [68], a Portuguese team proposed and tested the use of CNNs for semi-automated glioma segmentation in 2016 [69]. The literature continued to focus on semi-automated segmentation until early 2020, when a multi-institutional Chinese collaboration published the results of the first study on fully automated BM segmentation [70]. Many models were thenceforth developed; however, semi-automated segmentation retains the ideal balance between clinician re-/overview and the regional probability estimates. Automated algorithms can provide the former with information regarding BMs and are thus considered to be the optimal segmentation method for the time being [71].

### 3.2. Differential Diagnosis of Brain Metastases

While meticulous segmentation is essential, the more critical diagnostic challenge in brain metastases lies in their accurate differentiation from other intracranial lesions—including gliomas, PCNSLs, meningiomas, hemangioblastomas, cerebral abscesses, subacute infarcts, and post-treatment changes—as this distinction is pivotal for selecting the appropriate therapeutic strategy [72]. BMs are multifocal in up to 85% of hematogenous spread cases and are typically distinguished by their characteristic location (most commonly involve the corticomedullary junction) and spread pattern [73], although fungal infections and abscesses—especially in immunocompromised patients—can also present as multifocal lesions with ring enhancement on MRI [74]. Among the most challenging differential diagnoses is that of a solitary BM, which can radiologically mimic a high-grade glioma, both entities may present with ring enhancement, central necrosis, and significant peritumoral edema [75].

SVMs seem to be one of the best classifiers for distinguishing BMs from glioblastomas using post-contrast 3D T1-weighted gradient-echo MRI sequence radiomics, with mean accuracy, sensitivity, specificity, and area under curve (AUC) all ≥ 85% according to a 2019 study in over 400 patients [76]. A same-year, similar-sample study identified 13 classifiers with favorable predictive performances (AUC ≥ 95% and relative standard deviation; RSD ≤ 6%) and similarly observed the highest prediction efficacy (AUC = 90%) using SVMs. It is interesting to remark that the best classifier demonstrated superior clinical performance in terms of accuracy, sensitivity, and specificity compared to radiologists [77]. A more recent systematic review and meta-analysis on various ML models for BM differentiation from gliomas identified 16 studies with a pooled AUC of 91.6 ± 5.2%, a sensitivity of 86.8 ± 12.3% (16 studies), and a specificity of 84.3 ± 23.5% (15 studies) [78]. A 2023 comparison between ML and DL pipelines for BM and glioblastoma differentiation on multiparametric MRI including over 500 cases concluded that both pipelines achieved similar performance and that the peritumoral tissue is important for model refinement, just like the tumor itself [79].

Additionally, oxygen metabolic radiomics constitute a relatively recent and promising method of differential diagnosis between solitary BMs and glioblastomas that relies on oxygen metabolism assessment [80]. BMs exhibit lower proliferation rates and subsequently reduced oxygen demands compared to glioblastomas, engendering perceptible discrepancies in aerobic glycolysis, oxidative phosphorylation, and intratumoral tissue hypoxia [81]. Radiomic features associated with parameters such as tissue oxygen saturation (mitPO_2_) or the cerebral metabolic rate of oxygen (CMRO_2_) combined with 1D-CNNs have demonstrated superior diagnostic performance to radiologists [80]. A prospective feasibility study investigated preoperative multi-gradient echo and arterial spin labeling MRI sequences of BMs and glioblastomas using a combined Quantitative Susceptibility Mapping (QSM) and Quantitative Blood Oxygen Level-Dependent (qBOLD) model for phase and magnitude analysis, respectively [82]. The authors calculated three variables (oxygen extraction fraction, OEF; cerebral blood flow, CBF; and CMRO_2_) separately for the contrast-enhancing tumor (CET) and peritumoral non-enhancing T2 hyperintense region (NET2) using an artificial neural network (ANN). BMs displayed higher CET OEF (*p* = 0.03) values and CET/NET2 ratios for CBF (*p* = 0.04) and CMRO_2_ (*p* = 0.01) compared to glioblastomas. Additionally, all CET features were significantly higher than their NET2 counterparts for BMs (p = 0.01). An SVM combination of two features (CET OEF and CET/NET2 CMRO_2_) yielded the highest discriminative power, with an AUC of 94% and a diagnostic accuracy of 93%.

### 3.3. Non-Invasive Molecular Characterization

While differentiating BMs from other intracranial lesions is the first step in guiding treatment decisions, uncovering their molecular profile is also important for patient-specific pharmaceutical regimen tailoring. Traditional genomic characterization requires invasive stereotactic frame-based biopsies that are often challenging for multifocal or deep-seated lesions and carry a risk of mortality (0.7–4%) and morbidity (3–13%), such as neurological impairment and hemorrhage [83]. Ongoing AI model advancements and developments indicate that BM molecular classification can be achieved through non-invasive approaches as well, as BMs originating from different primaries demonstrate local environment differences and subsequently distinct radiomic features [68].

A study exploring the use of radiomic features from several MRI sequences showed significant classifier accuracy with an AUC ranging from 64% (NSCLC) to 82% (melanoma) and outperformed radiologists (especially in sensitivity for melanoma) [84]. Another study applying a transformer-based DL model to whole-brain MRI scans achieved an AUC of 87.8% in classifying BMs into five primary histology groups (lung, breast, melanoma, renal, and others) and emphasized the discriminative power of imaging data, although the technique included no external validation with an independent dataset [85]. In a study comparing 2D and 3D texture features extracted from T1-weighted post-contrast MRI sequences, a nested cross-validation approach demonstrated that 3D features—particularly with optimized gray-level quantization—offered superior classification performance. This was especially true for distinguishing brain metastases originating from lung cancer versus melanoma. However, differentiation between melanoma and breast cancer metastases remained less accurate [86]. Despite the promising results all the aforementioned studies, a 2024 retrospective multicenter German–American analysis of 545 metastases over an 18-year period included local and two external validation datasets and supported that the capability of MRI radiomics in predicting primary BM histology is actually limited [87]. The team additionally highlighted that classification performance improvement was not feasible using oversampling techniques and that a massive model performance overestimation can result from incorrect data partitioning.

**Figure 3:**
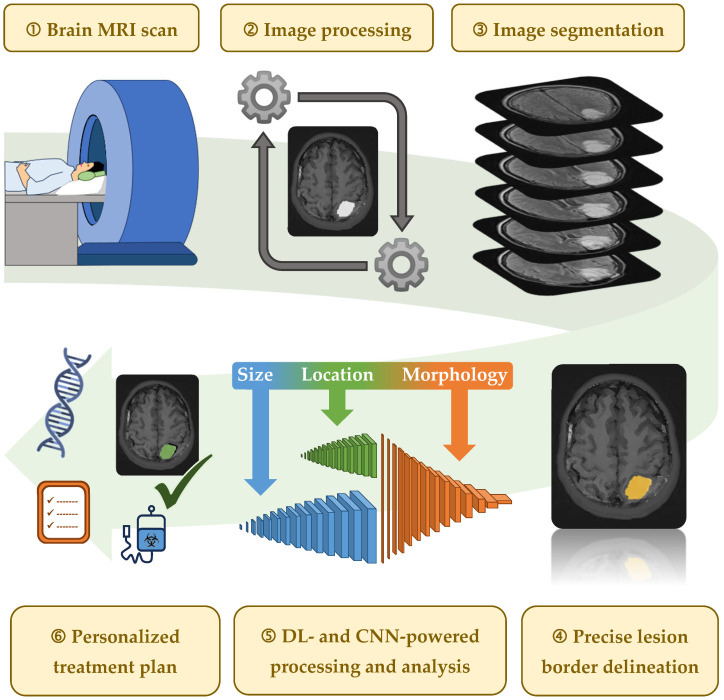
Step-by-step workflow depicting how AI-driven MRI-derived image processing, segmentation, and analysis contribute to precise lesion identification and patient-tailored treatment planning in neuro-oncology. AI = artificial intelligence; MRI = magnetic resonance imaging.

Figure 3 illustrates how AI facilitates personalized treatment planning in BM care.

## 4. AI-Assisted Therapeutic Planning

### 4.1. Surgical Planning and Intraoperative Assistance

Artificial intelligence is increasingly influencing the landscape of preoperative functional mapping, particularly in the context of brain metastases located near eloquent brain regions. AI-enhanced task-based functional MRI (fMRI) analysis improves the reliability and precision of functional localization by denoising BOLD signals (the signal while the patient is at rest—typically lying down with eyes closed) [88,89] and correcting motion artifacts [90]. In patients who are cognitively impaired or unable to cooperate with task-based paradigms, standard functional MRI techniques may be unreliable or infeasible. In such cases, resting-state fMRI (RS-fMRI) has emerged as a promising alternative, as it does not require active patient participation [91]. Recent advances have further enhanced RS-fMRI through the application of artificial intelligence, particularly deep learning models such as 3D CNNs. These models can automatically extract functional connectivity networks, enabling the identification of motor and language areas in patients with limited compliance [92,93]. Unlike task-based fMRI, RS-fMRI catches spontaneous low-frequency fluctuations in the BOLD signals. These intrinsic fluctuations may offer indirect insights into functionally connected networks such as the default mode network (DMN), motor network, or language network, offering a window into brain function without the need for active participation [88].

AI-based diffusion tensor imaging (DTI) and tractography further contribute by automating the reconstruction of key white matter tracts, using DL to improve anatomical accuracy even in the presence of tumor-induced distortion. DTI plays a central role in neurosurgical planning by enabling the visualization of white matter architecture, particularly in relation to lesions adjacent to eloquent tracts [94]. Conventional tractography methods rely on deterministic or probabilistic algorithms that use voxel-wise diffusion parameters (e.g., fractional anisotropy) and principal diffusion directions to reconstruct fiber pathways [95]. However, these methods often suffer from limitations such as false positives, failure in areas of complex fiber crossings, sensitivity to noise, and significant user dependency [96]. AI (and DL in particular) is being increasingly applied to overcome these limitations and enhance both the reliability and interpretability of tractography.

CNNs have demonstrated the ability to learn spatial–temporal diffusion patterns and improve bundle reconstruction fidelity. These models show promise in addressing challenges such as crossing fibers, partial volume effects, and abnormal anatomy caused by tumors or edema when compared to traditional techniques [97]. At the same time, AI-driven segmentation tools, such as U-Net models, provide precise delineation of metastatic tumors and perilesional edema, which is critical for combining structural and functional data. These elements are brought together through AI multimodal unification frameworks that fuse structural MRI, task-fMRI, RS-fMRI, and DTI data into patient-specific functional anatomical maps [98].

Additionally, predictive AI models based on preoperative imaging and clinical data are being developed to calculate the likelihood of postoperative deficits, helping surgical decision-making by balancing oncological goals with functional preservation. Finally, in cases where task-based imaging is not feasible, emerging AI approaches can synthesize task activation maps directly from RS-fMRI, providing a surrogate for functional localization and expanding access to individualized, network-preserving surgical planning [99]. Together, these AI-enhanced modalities represent a powerful toolkit for maximizing safety and precision in the neurosurgical management of metastatic lesions near eloquent brain regions.

In the context of neurosurgical planning for brain metastases, accurate segmentation plays a pivotal role in delineating lesion extent, estimating tumor volume, and guiding safe surgical corridors—particularly when lesions are adjacent to eloquent structures. While manual delineation remains common in clinical workflows, it is labor-intensive and subject to variability, especially in the presence of peritumoral edema or post-treatment changes [100]. DL-based segmentation tools, especially those utilizing CNN architectures, have emerged as practical aids by providing rapid, reproducible, and high-resolution delineations across multiple MRI sequences [101,102]. These models can distinguish the CET from surrounding edema and necrosis—information crucial for planning the extent of resection, anticipating brain shift, and co-registering structural data with functional imaging such as tractography or fMRI. Moreover, recent tools incorporate uncertainty quantification, offering confidence maps that help neurosurgeons gauge the reliability of the segmentation near critical areas [103]. 

Augmented reality (AR) enhances intraoperative precision by integrating this multimodal data into the surgical field. Together, AI and AR form a synergistic framework for individualized neurosurgical resection of brain metastases near eloquent areas. AR-assisted neurosurgical resection of brain metastases utilizes real-time 3D visualization of preoperative images, used in the surgical field via head-mounted displays (HMDs) or microscope-integrated heads-up displays (HUDs) [104]. This technology is useful in the delineation of tumor boundaries [105], adjacent critical cortical/subcortical structures (e.g., motor/speech areas) [106,107], and critical vascular structures, enhancing the surgeon’s spatial orientation during the operation. AR systems integrate frameless stereotaxy with automatic registration to align virtual models (segmented from preoperative imaging) to the patient’s anatomy, minimizing manual landmark-based registration errors [106].

Intraoperative AR updates, such as post-contrast T1-weighted MRI fusion, compensate for brain shift by dynamically adjusting the overlay to reflect tissue deformation during tumor debulking [108]. Key technical advantages include reduced reliance on monitor-based navigation (eliminating disruptive head-turning) and improved ergonomics by maintaining focus on the operative field [109]. However, limitations persist, including latency in AR projection refresh rates, registration inaccuracies in cases of severe brain shift (>5 mm), and dependence on preoperative imaging without real-time intraoperative updates unless paired with intraoperative MRI/CT [110]. Current systems like VIPAR (VIrtual Protractor with Augmented Reality) [111] or NeuroAR [106] emphasize workflow efficiency by projecting tumor boundaries and safety margins as color-coded holograms, though calibration demands meticulous attention to avoid misalignment. Future iterations aim to integrate AR with robotic platforms [112] and AI-driven predictive models to anticipate intraoperative anatomical changes.

### 4.2. Radiotherapy Planning Optimization

AI-driven dose planning for SRS on brain metastases utilizes advanced ML and DL algorithms to optimize target delineation, dose distribution, and critical structure sparing while reducing inter-operator variability and time-intensive manual planning [113]. Automated segmentation tools, such as U-Net and DenseNet architectures, enable precise tumor delineation by analyzing multimodal imaging (T1-weighted contrast-enhanced MRI, T2-FLAIR) to distinguish metastases from peritumoral edema or normal brain parenchyma and addressing challenges like lesion heterogeneity and irregular margins [113,114]. These models integrate radiomic features (texture, shape, intensity) to predict tumor biology and radiosensitivity, informing personalized dose prescriptions (e.g., margin adaptation based on infiltrative vs. circumscribed growth patterns).

Inverse optimization algorithms dynamically balance target coverage (≥95% planning target volume (PTV) prescription dose) with organ-at-risk (OAR) constraints (e.g., hippocampus, optic apparatus), generating non-isocentric, non-coplanar beam arrangements that achieve steep dose gradients (10–20% isodose line falloff) [115,116,117]. Real-time adaptation via onboard cone beam CT (CBCT) or MR-linac systems further refines delivery by compensating for intrafractional motion or anatomic shifts [116,117]. Challenges include validating generalizability across institutional imaging protocols, mitigating bias from underrepresented tumor subtypes in training datasets, and ensuring explainability of “black-box” DL decisions.

Emerging approaches explore the combination of generative adversarial networks (GANs)—a type of deep learning model capable of creating realistic synthetic images, such as CT scans—and reinforcement learning, which simulates decision-making over time based on predicted outcomes. Together, these techniques hold potential for developing adaptive fractionation strategies, where treatment dose and schedule could be adjusted in response to early imaging biomarkers indicating radiation necrosis or tumor recurrence [115,118]. Integration with molecular imaging (FET-PET, amino acid tracers) and diffusion tensor tractography [119,120] may further enhance biologically guided SRS planning.

Adaptive workflows leverage AI-driven tools like CBCT and ExacTrac stereotactic imaging to account for intrafractional motion and tumor volumetric changes, particularly in hypofractionated stereotactic radiosurgery (SRS) [117]. ML models also analyze longitudinal imaging to detect early treatment failure, allowing dose escalation or de-escalation based on tumor radiobiological response [121]. For radioresistant metastases, AI identifies optimal fractionation schemes (e.g., single- vs. multi-fraction SRS) by correlating genomic profiles with historical outcomes, mitigating risks of local progression seen in conventional fractionated approaches [122]. However, challenges persist in validating AI-generated plans against clinical endpoints, necessitating prospective trials to establish safety thresholds for AI-driven adaptive protocols [123].

### 4.3. AI-Driven Approaches to Systemic Therapy in BMs: Integrating Immunotherapy and Targeted Treatment

AI-driven radiomics has become a transformative tool for predicting systemic therapy response in brain metastases, especially for immunotherapy and targeted therapies. The extraction of MRI or CT images allows radiomics to non-invasively characterize tumor variety, microenvironment, and molecular profiles that correlate with treatment efficacy. For example, radiomic profiles linked to PD-L1 (programmed death ligand 1) expression, tumor-infiltrating lymphocytes (TILs), or EGFR mutation status (common in NSCLC-derived metastases) may predict response to anti-PD-1/PD-L1 inhibitors or EGFR tyrosine kinase inhibitors (TKIs) [124]. ML models trained on these features, combined with variables such as the neutrophil-to-lymphocyte ratio (NLR) and genomic profile, can distinguish between patients likely to benefit from immunotherapy and those requiring alternative treatments [125]. Studies suggest that tumors with radiomic evidence of peritumoral edema formation or vascularization after SRS may exhibit enhanced immune checkpoint inhibitor (ICI) penetration, improving distant brain control [126,127]. However, challenges remain, including the standardization of imaging protocols, validation across diverse groups, and integration with real-time liquid biopsy data [123,128]. Ongoing trials aim to refine these models to optimize combinatorial strategies (e.g., SRS and immunotherapy timing) and reduce neurotoxicity risks [127].

AI platforms can guide real-time treatment adjustments, such as optimizing TKI or ICI use based on evolving tumor characteristics. For EGFR-mutated NSCLC brain metastases, osimertinib (a third-generation EGFR-TKI) demonstrates CNS penetration and improved progression-free survival (PFS) compared to earlier TKIs, with AI models analyzing circulating tumor DNA (ctDNA) to adjust dosing [127,129]. ICIs such as pembrolizumab (anti-PD-1) are prioritized in high tumor mutational burden (TMB) (≥10 mutations/Mb) cases, where AI algorithms correlate spatial immune cell distribution on MRI with response durability [130,131]. Machine learning platforms synthesize DTI metrics, genomic subtyping (e.g., HER2/ALK status), and pharmacogenomic data to predict BBB permeability for chemotherapeutics like topotecan [115]. Adaptive neural networks also mitigate resistance by identifying clonal evolution patterns—critical in tumors with PI3K/AKT/mTOR pathway activation [132,133].

### 4.4. Emerging Approaches in Immunotherapy for BMs

ICIs have demonstrated effectiveness in subsets of patients with intracranial melanoma and NSCLC brain metastases, with retrospective analyses showing response rates comparable to systemic responses [134,135]. However, heterogeneity in tumor biology and the immunosuppressive CNS microenvironment impose advanced predictive tools. AI-driven platforms utilize radiomic features from MRI (e.g., peritumoral edema, enhancement patterns) and molecular data such as TMB and PD-L1 expression to identify patients likely to benefit from ICIs [123,131]. For example, ML models trained in multiparametric imaging and genomic data have identified radiogenomic signatures correlating with CD8+ T-cell infiltration and major histocompatibility complex I (MHC-I) expression, which predict ICI responsiveness [123].

AI-based models are increasingly utilized to refine immunotherapy selection in BMs by integrating imaging, molecular, and transcriptomic data. Radiomic profiling from MRI, when combined with biomarkers such as PD-L1 expression and TMB, has been used to predict responsiveness to ICIs [115,134]. More recently, spatial transcriptomics—mapping immune cell distribution within the TME—has identified “hot” versus “cold” immune phenotypes that correlate with ICI efficacy [131,135]. AI platforms trained on these data can stratify tumors by their immune landscape, enhancing precision in immunotherapy timing and choice. Additionally, emerging models utilize dynamic imaging changes on T2-FLAIR (fluid-attenuated inversion recovery) and diffusion-weighted MRI to distinguish pseudoprogression from true progression after immunotherapy, potentially minimizing premature treatment cessation [130]. Despite these advancements, challenges in the standardization of imaging protocols, biopsy sampling, and model validation across diverse populations remain [123,136,137].

**Figure 4:**
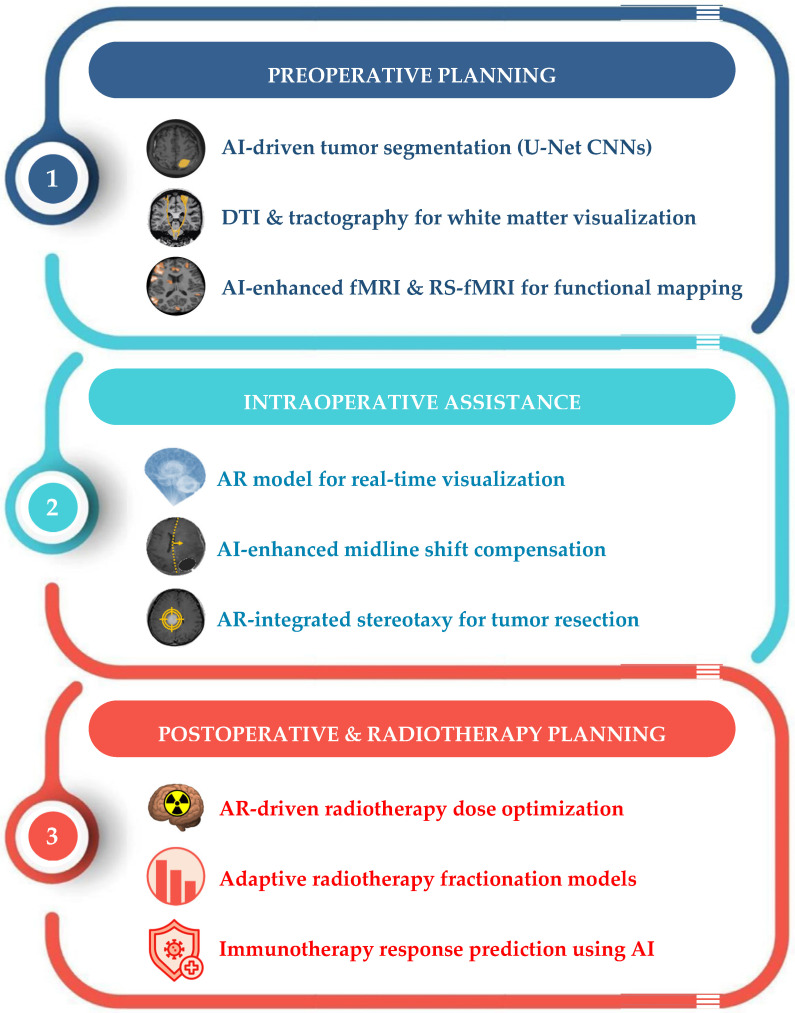
Linear flowchart summarizing how AI integrates different imaging and treatment modalities for preoperative planning, intraoperative guidance, and postoperative and radiotherapy planning in BMs. AI = artificial intelligence; AR = augmented reality; CNNs = convolutional neural networks; DTI = diffusion tensor imaging; fMRI = functional magnetic resonance imaging; RS-fMRI = resting-state functional magnetic resonance tomography.

Figure 4 summarizes the most important treatment-focused pre-, intra-, and post-operative applications of AI in BMs.

## 5. AI in BM Prognostic Assessment

### 5.1. Survival and Recurrence Prediction

AI can serve as a prognostic tool in BMs by combining data such as clinical, imaging, molecular, and treatment variables, to help clinicians make patient personalized therapeutic decisions. Traditional prognostic tools (such as the Graded Prognostic Assessment (GPA)) relying on clinical metrics like age, Karnofsky Performance Status, and extracranial disease lack the granularity to account for tumor heterogeneity, treatment response, or biomarkers. ML models—particularly Deep Neural Networks (DNNs)—address these limitations by analyzing multidimensional data to identify latent prognostic patterns that correlate with survival outcomes, such as radiomics on MRI (peritumoral edema, necrosis morphology) or genomic markers (EGFR/ALK alterations). For example, recent studies indicate that AI-driven survival models are superior (AUC > 0.85) compared to GPA (AUC ≅ 0.70) by combining DWI metrics, VTB, and systemic therapy timelines [115,138]. These models also enable real-time risk categorization, predicting early leptomeningeal disease progression or radiation necrosis susceptibility, thereby guiding personalized treatments [123,139].

AI is enhancing prognostic modeling in brain metastases by integrating radiomic, clinical, and molecular data into dynamic survival predictions. Unlike traditional tools like the GPA or Recursive Partitioning Analysis (RPA), AI models incorporate imaging features (e.g., tumor volume, edema) [101], clinical variables (e.g., Karnofsky Performance Status, systemic disease burden), and biomarkers (e.g., EGFR mutation status, PD-L1 expression, and TMB) [115,134]. Some approaches also include longitudinal data such as follow-up imaging or ctDNA to improve recurrence prediction [46,130]. These tools support tailored surveillance and treatment strategies, though broader adoption will require standardized imaging protocols, external validation, and prospective trials [136,137].

Awuah et al. highlighted the superiority of ANNs over traditional statistical methods in predicting the one-year survival rates; they have achieved higher accuracy by capturing nonlinear interactions between variables such as lesion multiplicity, peritumoral diffusion restriction, and genomic instability (increased tendency of the genome to mutations) [115]. Laufer and Bilsky emphasized the role of ML in correlating imaging phenotypes with molecular drivers (e.g., HER2 amplification in breast cancer metastases) to predict treatment response and PFS [140]. Furthermore, Staartjes et al. highlighted the clinical utility of ML-driven predictive analytics integrating tumor segmentation maps from contrast-enhanced T1-weighted MRI, metabolic PET data, and CSF cytokine profiles to stratify patients into risk groups for personalized adjuvant therapy planning [118]. Challenges remain in standardizing data acquisition (e.g., harmonizing DSC MRI protocols across institutions) and validating models prospectively [141].

Traditional prognostic indications for brain metastases recurrence risk after SRS and systemic therapy—such as the RPA and Score Index for Radiosurgery (SIR)—estimate the overall survival based on factors like the Karnofsky Performance Status, number and size of brain metastases, and extracranial disease control, but they are limited in their ability to predict local or distant recurrence following SRS and systemic therapy [142,143]. In contrast, recent ML models have introduced new approaches by enabling recurrence prediction through the integration of dynamic, patient-specific features such as tumor volumetrics, systemic therapy response patterns (TKIs, immunotherapy), and inflammatory biomarkers (NLR) [125,144]. For example, integrated models leveraging post-SRS serial MRI radiomics (textural heterogeneity, perilesional edema dynamics) and ctDNA profiles demonstrate superior accuracy in predicting early (<6 months) vs. late (>12 months) recurrence [118,145]. Key molecular determinants include EGFR/ALK alterations in NSCLC-derived metastases and HER2 status in breast cancer, which modulate BBB permeability and residual micrometastatic burden [146,147]. Challenges persist in the temporal calibration of these algorithms, particularly for patients receiving staggered or combination therapies (e.g., SRS with ICIs), where pseudoprogression and delayed radiation necrosis may confound imaging-based risk assessment [148,149]. Prospective validation of hybrid models combining ML-predicted recurrence risk with frailty indices (e.g., Risk Analysis Index) is ongoing to optimize surveillance intervals and salvage therapy timing [150].

### 5.2. Disease Progression Monitoring

AI-assisted tracking of new lesions and treatment response in brain metastases utilizes advanced computational techniques to analyze neuroimaging data, enabling precise quantification of tumor burden and therapeutic effect. DL algorithms, particularly CNNs, segment metastatic lesions on post-contrast MRI or CT with high accuracy, identifying subtle changes in size, morphology, and enhancement patterns that may escape the human eye [151]. Radiomics extracts multidimensional features, such as volume, shape, and perfusion kinetics, from imaging data, correlating these with molecular profiles (EGFR or HER2 status) and predicting early treatment resistance [152,153]. Automated platforms combine serial imaging with clinical data to produce predictive models for pseudoprogression versus true progression, a critical distinction in immunotherapy or targeted therapy [118,154]. For example, temporal radiomic signatures of perilesional edema or intralesional necrosis have shown utility in differentiating radiation necrosis from recurrent disease [123].

Following SRS or other radiation therapies, pseudoprogression (PsP; transient, inflammatory, or necrotic changes mimicking tumor growth) can be difficult to distinguish from true progression (TP) using conventional MRI. Recent studies have combined radiomics from multiparametric MRI—including DWI, perfusion-weighted imaging (PWI), and contrast-enhanced T1-weighted sequences—to train ML classifiers. For example, radiomic models analyzing texture, shape, and intensity heterogeneity within enhancing lesions have achieved diagnostic accuracies of up to 87% when validated against histopathology or longitudinal clinical outcomes [155]. These models often prioritize features such as reduced ADC values in TP versus PsP or altered perfusion metrics (relative cerebral blood volume [rCBV]) [156]. A meta-analysis highlighted the superiority of ML-driven approaches over conventional imaging alone, with a pooled sensitivity of 95.2% and a specificity of 82.4% [155]. DL architectures, such as CNNs, further enhance this by automating feature extraction from volumetric imaging data, capturing subtle spatial patterns imperceptible to human observers [157].

### 5.3. Longitudinal Follow-Up Strategies

AI-driven follow-up imaging protocols personalized to patient-specific recurrence risk in BMs integrate radiomics, ML, and clinical biomarkers to categorize patients into risk categories and enable personalized observation intervals and imaging modalities. These protocols combine quantitative imaging features (tumor volume, peritumoral edema, diffusion/perfusion parameters) with molecular data (EGFR mutation status, TTF-1/napsin A expression) and treatment history to predict recurrence likelihood or adverse radiation effects (AREs) after SRS [146,147]. For example, radiomics derived from post-SRS MRI can identify high-risk lesions prone to PsP or radiation necrosis, suggesting earlier advanced imaging (amino-acid PET or MR perfusion) [149,158]. ML models trained on longitudinal data may adjust follow-up frequency dynamically; low-risk patients (i.e., those with stable lesions with favorable biomarkers) transition to extended 6–12-month intervals, while high-risk groups (NSCLC with EGFR wild-type or rapid pre-treatment progression) undergo 3-month scans with multiparametric sequences [140,159]. This approach mitigates unnecessary imaging, limits excess healthcare costs, and facilitates early intervention for subclinical recurrences. Radiomics-based analysis of follow-up neuroimaging in BM patients offers a noninvasive means of assessing systemic progression risk. Among extracted imaging features, tumor volume, perilesional edema patterns, and hemorrhage on T1-weighted or FLAIR sequences have been associated with underlying molecular aggressiveness—such as EGFR mutations or high-antigen Kiel 67 (Ki-67) proliferation indices—that may correlate with extracranial tumor spread [147,160]. These insights support the use of AI-enhanced radiomics to guide personalized surveillance and systemic therapy decisions.

## 6. Challenges and Limitations of AI in BM Management

### 6.1. Data Standardization and Availability

Although AI is gradually and increasingly adopted in the clinical management of BMs, various obstacles limit its seamless integration. Chief among them is the—often significant—heterogeneity of datasets, which are typically derived from disparate sources that include multiple institutions (frequently headquartered in different countries), numerous MRI sequences, and diverse patient populations. If left unaddressed, this variability may introduce inconsistencies, omissions, or even within-data contradictions that complicate robust AI algorithm development. Such discrepancies additionally undermine the potential of the latter to generalize across diverse cohorts, subsequently restricting clinical applicability and diminishing reliability.

Another important challenge is the acquisition of extensive and correctly annotated datasets integrating clinical, radiological, and histopathological information for each patient. The scale and depth required for high-performance AI model training are frequently unachievable because the available datasets might lack sufficient samples. Moreover, data labeling is both laborious and financially inconvenient, hindering the availability of high-quality data for training. It is consequently necessary to establish standardized methodologies ensuring uniform data collection, consistent diagnostic guidelines, and structured data formatting. Fostering ethical and legal boundaries within multi-institutional collaborations would facilitate comprehensive dataset development, an indispensable prerequisite for training models able to accurately prognosticate in BMs. A further complication is associated with the origination of a significant amount of histopathological classification data from periods when BM molecular characterization was neither widespread nor prioritized, unintentionally introducing bias into training.

Recognizing the limited availability of diverse, high-quality datasets, the so-called “Brain Tumor Segmentation—Metastases (BraTS-METS) Challenge 2023” aimed to create a meticulously annotated dataset of untreated BMs across various MRI sequences from eight institutions for segmentation model development [161]. A total of 1303 scans were annotated through a stepwise process that combined initial AI-based segmentation, manual labeling by trained students, and expert review by neuroradiologists. Of these, 402 scans were made publicly available, while others were reserved for validation and testing purposes. Model accuracy was evaluated based on how well the AI matched expert annotations, with special attention given to minimizing both missed tumors and false positives. The best-performing algorithm demonstrated generally strong performance but showed considerable variability—particularly in identifying very small lesions, where accuracy dropped significantly. This initiative was important not only for creating a valuable resource for future research but also for exposing current limitations in AI models, especially their reduced reliability in detecting small brain metastases. It reinforced the need for better data standardization and algorithm refinement to support the safe clinical integration of AI-based tools in neuro-oncology [161].

### 6.2. Interpretability and the ‘Black Box’ Issue

Notwithstanding the noteworthy advancements in DL applications in BM imaging, such systems often function intricately and non-transparently in terms of decision-making rationale. This immanent uncertainty encourages doubt among clinicians, who are hesitant to fully adopt AI-driven models into their practice, especially in critical fields such as BM management. The main obstacle is the lack of clear understanding, as AI models often make predictions without explicitly clarifying the factors driving their conclusions, decisions, and suggestions.

Though challenging, endeavors to clarify AI-generated predictions and improve model transparency are ongoing. The rise of explainable AI (XAI) has provided understandable insights into their decision-making process and enhanced explainability, for instance regarding how AI differentiates between BM recurrence and post-SRS radionecrosis [162]. Rendering model decisions easier to interpret allows clinicians to feel more confident in AI-generated recommendations and more smoothly integrate their insights into everyday clinical decision-making. Of course, achieving full clarity remains a tough obstacle—which will probably not be overcome anytime soon—given the reliance of DL systems on a vast number of parameters that complicate the pinpointing of exact factors influencing individual predictions.

### 6.3. Ethical and Legal Considerations

Beyond data availability and interpretability issues, the ethical consequences of AI integration into BM care raise major concerns, especially regarding patient data privacy. Since AI systems depend on enormous amounts of sensitive information—including medical history, genetic data, and diagnostic results—maintaining well-fortified data security across different platforms and institutions is particularly challenging, as any leak or unauthorized access directly threatens patient confidentiality. Additionally, the risk of bias in AI-powered prediction models further aggravates ethical issues; if the datasets used for training overrepresent specific demographic groups while underrepresenting others, there is a risk of unbalanced predictions that engender discrepancies in diagnostic accuracy and treatment plan recommendations. To prevent these issues, strict regulations ought to be put in place to ensure informed consent, improve data usage clarity, and maintain high security standards.

From a legal perspective, the problem of responsibility and accountability when AI-based models produce incorrect diagnoses or misleading treatment plans remains unresolved. The question of legal liability differs across regions and continues to constitute a major challenge as AI is more widely adopted in medical day-to-day practice. For the time being, the rules governing AI use in healthcare are neither strict nor stringent enough, with many such systems lacking consistent efficiency—and sometimes even safety—standards. It is consequently crucial for respective regulatory bodies to establish unambiguous guidelines governing the approval, regulation, and deployment of AI applications to safeguard patient welfare and cultivate both clinician and patient trust in AI-assisted BM management.

## 7. Future Directions and Innovations in AI for BMs

As AI becomes more embedded in neuro-oncology, it is advancing the management of brain metastases through data-driven personalization and adaptive therapeutic planning. Advanced AI algorithms are increasingly capable of synthesizing high-dimensional datasets, including high-resolution neuroimaging (e.g., DTI, perfusion metrics), tumor genomic profiles, and clinical biomarkers, to generate predictive models for treatment response and recurrence risk [115,163]. Real-time intraoperative AI systems incorporating intraoperative MRI and electrophysiological mapping data can dynamically adjust resection boundaries to minimize injury to eloquent cortex while maximizing cytoreduction, particularly in BMs infiltrating sensorimotor or language pathways [115,164]. In radiosurgery, AI-driven platforms may soon automate dose painting by integrating real-time O-arm imaging with tumor radiobiology (e.g., hypoxia signatures, repair capacity) to optimize SRS plans during fraction delivery [148,165]. For precision medicine, neural networks trained on multiomic datasets (e.g., methylation profiles, proteomics) could identify patient-specific vulnerabilities—such as HER2/neu amplification in breast cancer metastases or ALK fusions in NSCLC—and enable tailored immunotherapy or small-molecule regimens [163,166]. Future research must prioritize prospective AI-derived decision pathway validation in multicenter trials, with an emphasis on treatment stratification based on molecular subtypes (e.g., radioresistant vs. radiosensitive metastases) and quality-of-life preservation [167,168]. Ethical frameworks for AI deployment, including bias mitigation in underrepresented genomic cohorts and explainability in black-box models, remain critical to clinical translation. Ultimately, the convergence of AI with emerging modalities—such as cerebral organoid-based drug testing and nanoparticle-enhanced brachytherapy—promises to transform brain metastasis care from reactive palliation to proactive, biologically rationalized intervention [168].

## 8. Conclusions

The ongoing integration of AI into BM management can facilitate the execution of cumbersome work and streamline persisting diagnostic and therapeutic issues with the help of ML and DL. Multiple AI applications are already approaching daily clinical practice implementation, especially regarding lesion detection, border delineation, and differentiation between BMs and gliomas with superhuman accuracy. At the same time, radiotherapy planning and dose distribution, safer resection support near eloquent brain areas intraoperatively, as well as post-treatment models aiding in the distinction of true progression from pseudoprogression, combined with more accurate survival and recurrence assessment, are also nearing clinical maturity.

Although the above developments have the potential to enhance clinical decision-making and thereby ameliorate patient outcomes, important challenges including data availability, transparency, and ethical/legal considerations limit their applicability and wide adoption. Future endeavors should hence prioritize the development of generalizable AI models through the acquisition of large and diverse datasets, the combination of multimodal imaging and molecular information, as well as external validation. Once the above standards are met, BM management can be gradually transformed into much more efficient and patient-tailored care.

## Data Availability

Not applicable.

## Abbreviations

The following abbreviations are used in this manuscript:

ADC	Apparent diffusion coefficient
AI	Artificial intelligence
AKT	Protein kinase B
ALK	Anaplastic lymphoma kinase
ALL-IDB	Acute Lymphoblastic Leukemia Image Database
ANN	Artificial neural network
AR	Augmented reality
AREs	Adverse radiation effects
AUC	Area under the curve
BBB	Blood–brain barrier
BM	Brain metastasis
BOLD	Blood oxygen level-dependent
BraTS-METS	Brain Tumor Segmentation—Metastases
CBCT	Cone beam CT
CBF	Cerebral blood flow
CET	Contrast-enhancing tumor
CMRO2	Cerebral metabolic rate of oxygen
CNNs	Convolutional neural networks
CNS	Central nervous system
CSF	Cerebrospinal fluid
ctDNA	Circulating tumor DNA
DDSM	Digital Database for Screening Mammography
DL	Deep learning
DMN	Default mode network
DNNs	Deep neural networks
DSC	Dynamic susceptibility contrast
DTI	Diffusion tensor imaging
DWI	Diffusion-weighted imaging
EGFR	Epidermal growth factor receptor
ESP-Unet	Edge Strengthening Parallel Unet
FDA	Food and Drug Administration
FET	O-(2-[18F]fluoroethyl)-L-tyrosine ([18F]FET)
FLAIR	Fluid-attenuated inversion recovery
fMRI	Functional MRI
FRT	Fractionated radiotherapy
GAN	Generative adversarial networks
GPA	Graded Prognostic Assessment
HER2	Human epidermal growth factor receptor 2
HMD	Head-mounted displays
HUD	Head-up displays
IBM	International Business Machines Corporation
ICI	Immune checkpoint inhibitor
IQ-OTH	Iraq Oncology Teaching Hospital
MHC	Major histocompatibility complex
mitPO2	Tissue oxygen saturation
ML	Machine learning
MMPs	Matrix metalloproteinases
MRI	Magnetic resonance imaging
mTOR	Mammalian target of rapamycin
NCCD	National Center for Cancer Diseases
NET2	Non-enhancing T2 hyperintense region
NLP	Natural language processing
NSCLC	Non-small cell lung cancer
NYU	New York University
NYUMets	NYU Langone Health database
OAR	Organ at risk
OCT	Optical coherence tomography
OEF	Oxygen extraction fraction
PCNSL	Primary central nervous system lymphoma
PD-1	Programmed cell death protein 1
PD-L1	Programmed cell death ligand 1
PFS	Progression-free survival
PI3K	Phosphoinositide 3-kinases
PsP	Pseudoprogression
PTV	Planning target volume
qBOLD	Quantitative blood oxygen level-dependent
QSM	Quantitative susceptibility mapping
rCBV	Relative cerebral blood volume
RDD	Research Data Deposit
RPA	Recursive partitioning analysis
RSD	Relative standard deviation
RS-fMRI	Resting-state fMRI
SIR	Score Index for Radiosurgery
SRS	Stereotactic radiosurgery
SVM	Support vector machines
TIL	Tumor-infiltrating lymphocytes
TKI	Tyrosine kinase inhibitor
TMB	Tumor mutational burden
TME	Tumor immune microenvironment
TP	True progression
UCSF-BMSR	University of California San Francisco Brain Metastases Stereotactic Radiosurgery
VEGF	Vascular endothelial growth factor
XAI	Explainable AI
